# Value of tyrosinase RNA detection by an RT–PCR method in melanoma prognosis

**DOI:** 10.1038/sj.bjc.6600834

**Published:** 2003-03-18

**Authors:** K Konstantopoulos

**Affiliations:** 1 University of Athens School of Medicine, First Department of Medicine at Laikon Hospital, Athens 11527, Greece

**Sir**,

The paper published in *Br J Cancer* (2002, **87**: 181–186) on the prognostic significance of molecular detection of melanoma cells in melanoma patients under adjuvant interferon (Gogas *et al*, 2002) seems to be a continuation of the protocol we first applied in the same research laboratory utilizing the same facilities, the same methods and at least the same of some reagents (e.g. the ATTCC SK-mel-28 cells) we used in an analogous work. The results of this work have already been published (Konstantopoulos *et al*, 2001).

Gogas *et al* give no information pertaining to the status of melanoma cells circulating in their patients before any surgical manipulation (i.e. at first presentation). In our work, in 27 patients tested, one was already positive before operation becoming negative some time following operation. No data on that point are given on the 418 cases studied by Gogas *et al*. This type of information may permit further confirmation of a theory claiming that surgery manipulations may cause forced release of melanocytes to blood circulation (Foss *et al*, 1995).

Gogas *et al* claim that no evidence for an interference of the so-called ‘illegitimate transcription’ (Chelly *et al*, 1989) was shown in normal samples; this is in agreement with our observations as well. However, the possibility for the induction of transcription of tyrosinase gene under interferon administration should not be neglected. Interferon is a known inducer of expression of genes coding for several molecules, the best example being several cell surface markers. For a more reliable conclusion to be drawn, one should also include subjects under similar doses of interferon for other purposes as controls.
